# Host Stress Signals Stimulate Pneumococcal Transition from Colonization to Dissemination into the Lungs

**DOI:** 10.1128/mBio.02569-21

**Published:** 2021-10-26

**Authors:** Fayez Alghofaili, Hastyar Najmuldeen, Banaz O. Kareem, Bushra Shlla, Vitor E. Fernandes, Morten Danielsen, Julian M. Ketley, Primrose Freestone, Hasan Yesilkaya

**Affiliations:** a Department of Respiratory Sciences, University of Leicester, Leicester, United Kingdom; b Department of Biology, College of Science, Majmaah University, Majmaah, Saudi Arabia; c Department of Biology, College of Science, University of Sulaimani, Sulaymaniyah, Iraq; d Department of Biology, College of Science, University of Mosul, Mosul, Iraq; e MS-Omics ApS, Frederiksberg, Denmark; f Department of Genetics and Genome Biology, University of Leicester, Leicester, United Kingdom; Albert Einstein College of Medicine

**Keywords:** bacterial infection, *Streptococcus pneumoniae*, stress hormone, two-component regulatory system, virulence

## Abstract

Streptococcus pneumoniae is an asymptomatic colonizer of the nasopharynx, but it is also one of the most important bacterial pathogens of humans, causing a wide range of mild to life-threatening diseases. The basis of the pneumococcal transition from a commensal to a parasitic lifestyle is not fully understood. We hypothesize that exposure to host catecholamine stress hormones is important for this transition. In this study, we demonstrated that pneumococci preexposed to a hormone released during stress, norepinephrine (NE), have an increased capacity to translocate from the nasopharynx into the lungs compared to untreated pneumococci. Examination of NE-treated pneumococci revealed major alterations in metabolic profiles, cell associations, capsule synthesis, and cell size. By systemically mutating all 12 two-component and 1 orphan regulatory systems, we also identified a unique genetic regulatory circuit involved in pneumococcal recognition and responsiveness to human stress hormones.

## INTRODUCTION

Streptococcus pneumoniae is one of the most common causes of community- and hospital-acquired pneumonia as well as otitis media, meningitis, and septicemia (1). In addition to its pathogenic potential, the pneumococcus harmlessly colonizes the upper respiratory tract in approximately 10 to 40% of healthy adults and children, a state known as carriage (1). Despite extensive studies of pneumococcal carriage and infection, fundamental aspects of the shift from colonization to translocation into the lungs are not fully understood, but we believe that host hormonal signals may be involved in this transition.

In the human host, microbes encounter a variety of chemical signals that can fundamentally affect their behavior, the most studied of which are the catecholamine stress hormones dopamine (Dop), epinephrine (Epi), and norepinephrine (NE) (2). The microbes with pathogenic potential are known to sense the infection response status of their host by monitoring hormone levels, particularly those associated with stress (3). The most widely accepted mechanism by which stress hormones exert their effect on microbes is through their influence on iron metabolism and growth. The catechol moiety within the stress hormones can directly complex with the ferric iron of the host iron-sequestering protein transferrin (Tf) or lactoferrin (Lf) (4). This complex weakens Fe binding to Tf and Lf through reduction of ferric Fe, enabling uptake by bacteria and growth in serum or blood (4). Catecholamines also directly affect bacterial virulence and biofilm formation (2–4). Given the complex nature of stress hormone-bacterium interactions, it is highly plausible that there are additional unrecognized mechanisms involved in microbial responses to stress hormones.

Bacteria have developed interkingdom cross-signaling mechanisms to sense and process stress hormone signals. The QseBC two-component system (TCS) modulates virulence gene expression in enterohemorrhagic Escherichia coli (5, 6) and is conserved among several Gram-negative bacterial species. QseC, a membrane-bound sensor histidine kinase, is proposed to be an adrenergic receptor that phosphorylates the QseB response regulator (5). Moreover, QseE has been suggested to be a second histidine kinase adrenergic receptor and transduces a signal to QseF as part of another TCS (6). QseE senses multiple signals, including Epi, sulfate, and phosphate. Interference with the bacterial stress hormone-sensing mechanisms has been suggested to be a viable strategy for devising novel anti-infectives (7). It was demonstrated by structure-activity relationship studies that QseC function can be selectively modified using a prodrug, LED209, that impairs QseC function by allosterically modifying lysines in the sensor, preventing the activation of the virulence cascades of several Gram-negative pathogens both *in vitro* and *in vivo* (7). However, there is now doubt that QseC and QseE are adrenergic receptors as Pullinger et al. demonstrated that mutation of these in Salmonella did not abolish NE or Epi responsiveness (8). While the impact of stress hormones on Gram-negative bacteria has been well studied, knowledge on stress hormone responses in Gram-positive bacteria is currently limited.

Stress is recognized as an important risk factor for the progression of pneumococcal diseases. Epidemiological studies established that the incidence of pneumococcal infections increases among individuals living in poverty or poor living conditions (9, 10). In addition to exposure to endogenously produced stress hormones, the pneumococcus is exposed to catecholamine stress hormones as inotropic agents, which are widely used to maintain heart and kidney function in acutely ill patients (11), who are well recognized to be at risk of pneumococcal infection. Indeed, stress experienced by the elderly, hospitalized patients, and the immunocompromised correlates with an increased risk of pneumococcal infection (12). More direct evidence comes from the fact that plasma stress hormone levels in patients with pneumococcal pneumonia have been detected to be significantly higher than those in healthy individuals (13). Additionally, in an experimental mouse model of pneumococcal pneumonia, mice preexposed to stress are more susceptible to primary and secondary pneumococcal infections (14).

The clinical relevance of stress and stress hormones for pneumococcal infection has been experimentally verified recently. We previously demonstrated that S. pneumoniae is stress hormone responsive and that therapeutic levels of norepinephrine both increase pneumococcal growth and alter the expression of genes involved in metabolism and virulence (15). In other work, pneumococci were shown to recognize multiple host factors such as glucose, cell lysates, ATP, and NE as signals to disperse from biofilms (16). However, the molecular mechanisms by which catecholamine signals are recognized and processed by pneumococci are unknown. Here, we describe how the pneumococcus recognizes host stress hormones and characterize the physiological responses occurring within the pneumococcus. Using a well-established mouse model of pneumococcal colonization, we found that NE treatment enhances the pneumococcal transition from epithelial colonization to dissemination into the lungs. The regulatory cascade responsible for the recognition and processing of stress hormone signals in the pneumococcus was identified, the first such system described in Gram-positive bacteria.

## RESULTS

### Norepinephrine increases S. pneumoniae migration into the lungs.

**FIG 1:**
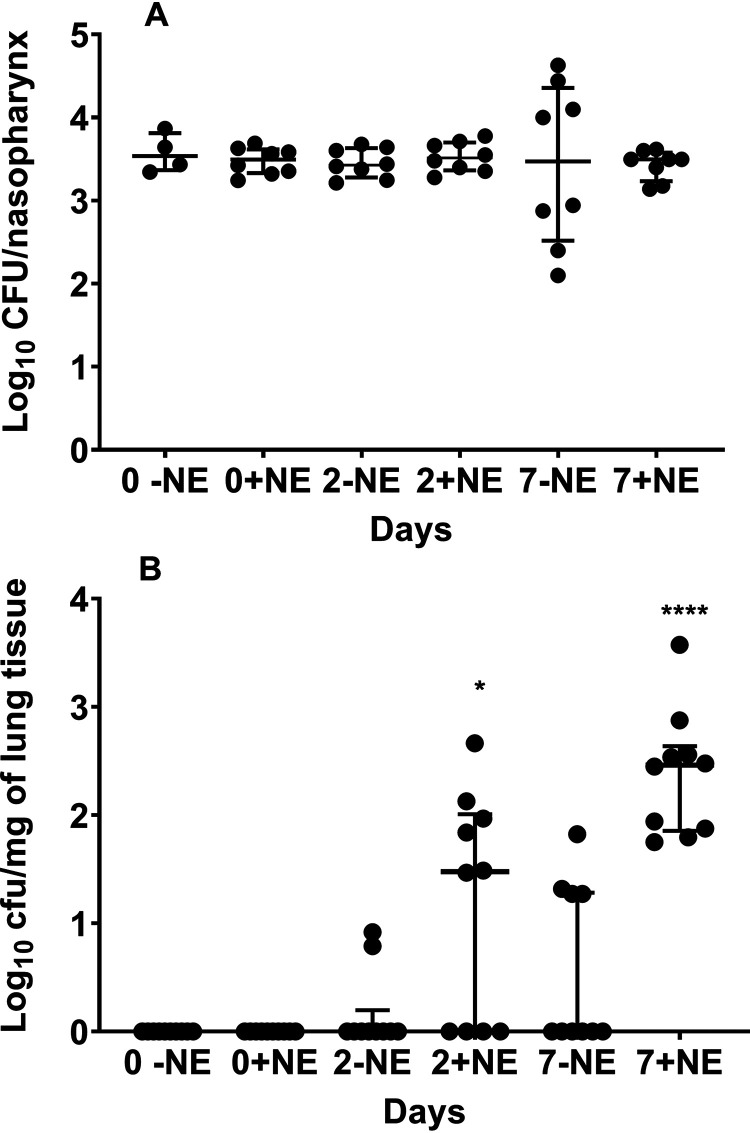
Norepinephrine (NE) increases pneumococcal translocation from the nasopharynx into the lungs. Mice were inoculated intranasally with approximately 5 × 10^5^ CFU of the type 2 D39 strain treated (+) or not (−) with 50 mM NE. Nasopharyngeal (A) and lung (B) bacterial loads were determined immediately after infection (day 0) or 7 days after infection. Each dot represents the CFU count in an individual mouse. The data were analyzed using the Mann-Whitney test. The median colony counts are shown with a horizontal line, while the vertical lines depict interquartile ranges. ****, *P* < 0.0001.

To test the hypothesis that exposure to host stress hormones increases S. pneumoniae dissemination from the nasopharynx into the lungs, we utilized a mouse model of pneumococcal colonization. In this model, nasal inoculation results in pneumococcal carriage in the nasopharynx without any sign of disease for up to 4 weeks. Therefore, compared to nontreated bacteria, growth in the presence of NE would result in higher levels of pneumococci in the lungs if NE affects pneumococcal infectivity. One hour after infection, the bacterial loads in the nasopharyngeal wash samples were the same for mice inoculated with nontreated D39 pneumococci and those inoculated with pneumococci grown in the presence of NE overnight, and no pneumococci were detected in bronchoalveolar lavage (BAL) fluid samples in either the test or control cohorts (Fig. 1A and B). In contrast, at 7 days postinfection, while the bacterial counts in the nasopharynx were similar between the test and control groups, prior growth of D39 pneumococci with NE resulted in more bacterial dissemination to the lungs than in the control group (*P* < 0.01) (Fig. 1A and B). To determine whether the observed impact of NE treatment is pneumococcal strain specific, we repeated the experiment with a serotype 4 TIGR4 strain (see Fig. S1 in the supplemental material). We obtained results for strain TIGR4 similar to those demonstrated for the type 2 D39 strain, showing that NE-mediated *in vivo* events are reproducible with another serotype. In contrast to growth overnight with NE, with exposure to the catecholamine for only 30 min before intranasal instillation, nasopharyngeal numbers of pneumococci were similar to those of the untreated control culture (Fig. S2), and no pneumococci could be recovered from the lungs (data not shown). These results suggest that longer exposure to NE increases S. pneumoniae infectivity.

10.1128/mBio.02569-21.1 FIG S1 Norepinephrine increases the translocation of TIGR4 from the nasopharynx into the lungs. Mice were inoculated intranasally with approximately 5 × 10^5^ CFU TIGR4 pneumococci treated (+) or not (−) with 50 μM norepinephrine (NE) in 20 μl PBS (pH 7.0). Nasopharyngeal (A) and lung (B) bacterial loads were determined immediately after (day 0) and 2 and 7 days after infection. Each dot represents the CFU count in individual mice. The data were analyzed using the Mann-Whitney test. The median colony counts are shown with a horizontal line, while the vertical lines depict interquartile ranges. *, *P* < 0.05; ****, *P* < 0.0001. Download FIG S1, TIF file, 0.6 MB. Copyright © 2021 Alghofaili et al. 2021 Alghofaili et al. https://creativecommons.org/licenses/by/4.0/ This content is distributed under the terms of the Creative Commons Attribution 4.0 International license.

10.1128/mBio.02569-21.2 FIG S2 A short period of norepinephrine treatment (30 min) does not affect pneumococcal numbers in the nasopharynx. Mice were inoculated intranasally with pneumococci (5 × 10^5^ CFU) treated (+) or not (−) with norepinephrine (NE). Download FIG S2, PDF file, 0.2 MB. Copyright © 2021 Alghofaili et al. 2021 Alghofaili et al. https://creativecommons.org/licenses/by/4.0/ This content is distributed under the terms of the Creative Commons Attribution 4.0 International license.

### Norepinephrine-induced pneumococcal gene expression profile *in vivo*.

To determine the mechanism underlying increased dissemination from the nasopharynx into the lungs induced by stress hormone exposure, we evaluated the expression of selected genes that have previously been shown to be differentially expressed in NE-treated pneumococci *in vitro* relative to pneumococci recovered from the nasopharynx. Similar to what was found *in vitro* (15), at 7 days postinoculation, the changed gene expression pattern associated with NE treatment was also seen *in vivo* with nasopharyngeal pneumococci (Table 1). However, there were exceptions. For example, while *nanB*, *galK*, and *ritR* expression was upregulated *in vitro*, *in vivo*, the increase in the expression of these genes did not change. Similarly, *spxB* expression was downregulated *in vitro*, whereas *in vivo*, *spxB* expression increased significantly in the nasopharynx. These exceptions in gene expression changes likely reflect specific differences between *in vitro* and *in vivo* environments.

**TABLE 1:** *In vivo* expression of selected genes in NE-treated S. pneumoniae relative to expression without NE^*a*^

Target	Function	Fold change in expression (SD)
*rgg144* (SPD_0144)	Transcriptional regulator	2.4 (0.16)
*rgg939* (SPD_0939)	Transcriptional regulator	3.6 (0.3)
*ritR* (SPD_0344)	DNA-binding response regulator	0.9 (0.14)
*strH* (SPD_0063)	*N*-Acetyl hexosaminidase	4.2 (0.22)
*bgaC* (SPD_0065)	β-Galactosidase	3.4 (0.3)
*nanB* (SPD_1499)	Neuraminidase B	1.3 (0.14)
*nanA* (SPD_1504)	Neuraminidase A	3.9 (0.35)
*piuA* (SPD_1652)	Iron compound ABC transporter	3.7 (0.20)
*pflB* (SPD_0420)	Pyruvate formate lyase	0.4 (0.1)
*spxB* (SPD_0722)	Pyruvate oxidase	2.7 (0.2)
*galK* (SPD_1634)	Galactokinase	1.3 (0.28)
*cps2A*	Capsule synthesis	0.6 (0.14)

a Relative expression was calculated from 2 independent experiments, and standard deviations are indicated in parentheses. The expression of target genes was normalized to the housekeeping gene *gyrB*.

### Norepinephrine treatment alters pneumococcal morphology and capsule synthesis.

**FIG 2:**
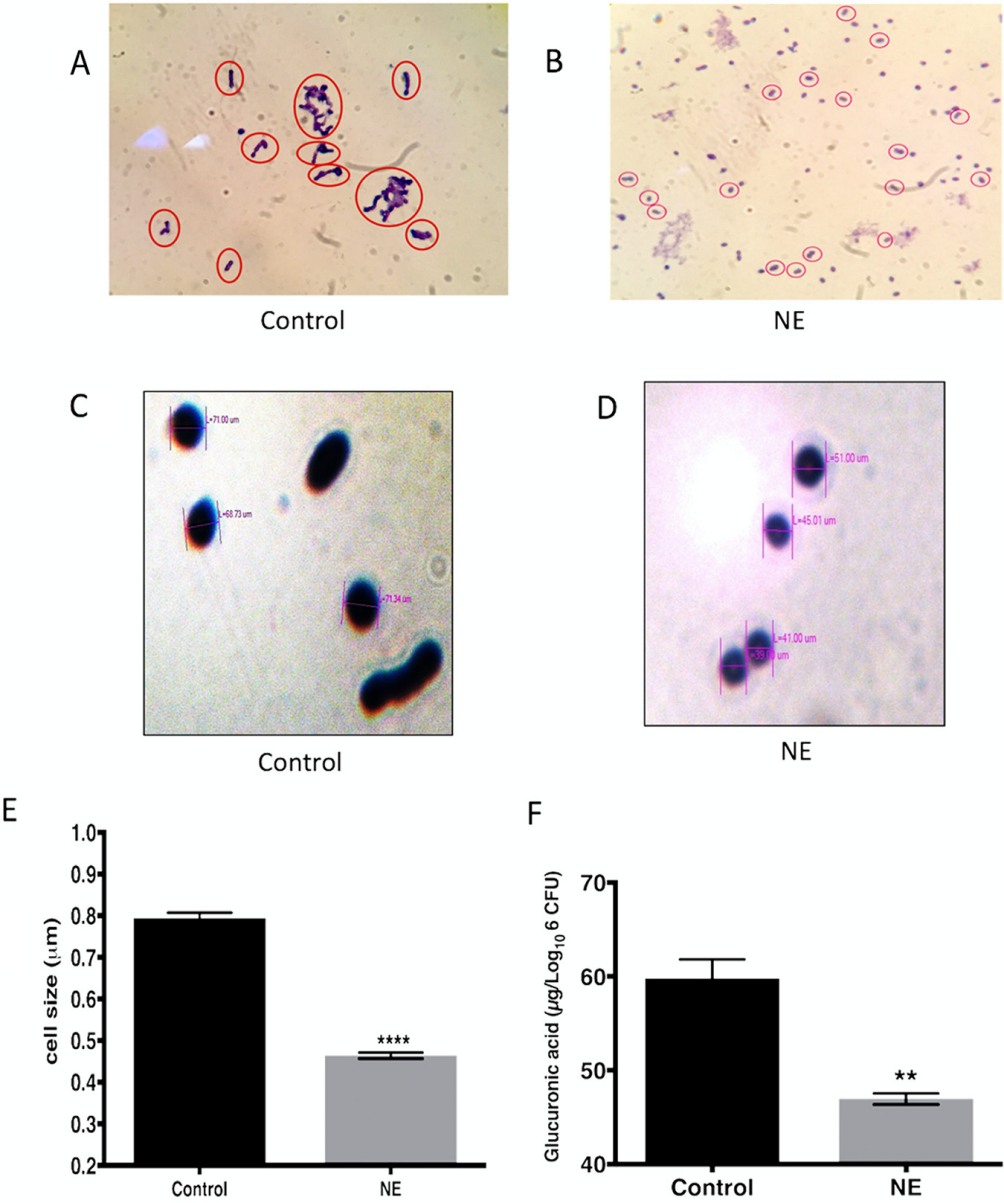
Phenotypic characterization of norepinephrine-exposed pneumococci. (A and B) Light microscopy images of Gram-stained S. pneumoniae strain D39 (magnification, ×100) after growth overnight in serum-SAPI medium with no additions (A) or growth in serum-SAPI medium in the presence of 50 mM NE (B). Red circles indicate the localization of pneumococci (*n* = 3). (C and D) Analyses of S. pneumoniae strain D39 cell size after growth overnight in serum-SAPI medium with no additions (C) or in the presence of 50 mM NE (D) (*n* = 80). An unpaired *t* test was used for cell size data comparisons. (E) Mean sizes of 80 cells counted from the cultures shown in panels C and D. (F) Histogram showing the capsule contents of the cultures shown in panels C and D (*n* = 3).

We examined the effect of NE on the morphology of the pneumococcus. An examination of NE-exposed cultures showed that S. pneumoniae cell morphology is distinct from that of nonsupplemented control cultures. Cell-to-cell associations differed, with short chains in control cultures compared to single cells or diplococci in NE-treated cultures (Fig. 2A and B). Furthermore, pneumococcal cell size was significantly reduced after NE treatment (0.46 μm ± 0.007 μm; *n* = 80) compared to controls (0.79 μm ± 0.013 μm; *n* = 80) (*P* < 0.0001) (Fig. 2C to E).

To further investigate if the NE-induced cell size reduction was due to a decrease in capsule synthesis, glucuronic acid was quantified as an indicator of pneumococcal capsular polysaccharide in control and stress hormone-treated cultures (17). Consistent with the cell size reduction, NE-treated pneumococci had significantly lower glucuronic acid levels (46.94 ± 0.5952 μg/10^6^ CFU; *n* = 3) than untreated pneumococci (59.80 ± 2.018 μg/10^6^ CFU; *n* = 3) (*P* < 0.01) (Fig. 2F). Collectively, these results suggest that there is an association between NE-induced reductions in cell size and capsular polysaccharide synthesis.

Next, we investigated how NE exposure led to a reduction in pneumococcal capsule synthesis. It is known that the Leloir galactose catabolic pathway is involved in capsule synthesis in S. pneumoniae as the intermediates generated by the catabolism of galactose act as precursors for capsule synthesis (18). We hypothesized that the reduction in capsule synthesis is due to NE repression of *galK*, a key gene in the Leloir pathway that codes for a galactokinase. To determine if NE suppresses the expression of *galK*, we constructed a reporter strain in which *lacZ* expression was driven from the *galK* promoter (P*galK*::*lacZ*-wt). No induction of β-galactosidase activity in the presence of 50 μM NE was observed, indicating that the NE-induced reduction in capsule synthesis is not due to suppression of *galK* transcription (Fig. S3).

10.1128/mBio.02569-21.3 FIG S3 The addition of 50 μM NE does not induce the promoter of the galactokinase gene (*galK*). The β-galactosidase activity level of the P*galK*::*lacZ*-wt reporter strain in the presence (+) or absence (−) of 50 μM NE was determined. Activity is expressed in Miller units per 10^7^ CFU (nanomoles of *p*-nitrophenol per minute per milliliter). Error bars indicate the SEM. Values are the averages from three independent experiments, each with three replicates. Download FIG S3, PDF file, 0.3 MB. Copyright © 2021 Alghofaili et al. 2021 Alghofaili et al. https://creativecommons.org/licenses/by/4.0/ This content is distributed under the terms of the Creative Commons Attribution 4.0 International license.

### Impact of NE on the pneumococcal metabolome.

To characterize the pneumococcal metabolic response to NE, we characterized the extracellular metabolites of stress hormone-treated cultures. A total of 34 metabolites identified by chemical standards, 21 metabolites annotated by mass spectrometry (MS) spectral library matching, and 17 unknown metabolites were measured (Data Set 1). The growth medium was included as a control, and it was observed that several metabolites appeared to be produced during growth, e.g., proline, glutamic acid, phenylalanine, aspartic acid, ketoleucine, fumarate, malate, lactic acid, and several unknown metabolites. Although NE treatment produced only small differences, the NE-treated cells appeared to have higher activity/growth than the control as more of the individual metabolites were consumed and a larger amount of fumarate was present.

10.1128/mBio.02569-21.7 DATA SET S1 Metabolome analysis of NE-treated S. pneumoniae. Data are derived from 3 independent experiments. The analysis was done by MS-Omics (Frederiksberg, Denmark). Download Data Set S1, XLSX file, 0.09 MB. Copyright © 2021 Alghofaili et al. 2021 Alghofaili et al. https://creativecommons.org/licenses/by/4.0/ This content is distributed under the terms of the Creative Commons Attribution 4.0 International license.

The intracellular metabolites varied more than the extracellular samples between the control and NE-treated pneumococci. However, data normalization to the sum of all metabolites reduced the observed differences between the two groups. A total of 33 identified, 31 annotated, and 21 unknown metabolites were measured. Fumarate, lactic acid, and phosphoenolpyruvate levels were significantly lower following NE treatment than in the control when the data were analyzed with a *t* test using Benjamini-Hochberg correction and a false discovery rate of 10%. These compounds are associated with primary metabolism, particularly glycolysis, and their lower levels are consistent with NE induction of more rapid growth as reflected in a higher energy requirement and enhanced carbon consumption.

### TCS09 is involved in pneumococcal catecholamine recognition and processing.

**FIG 3:**
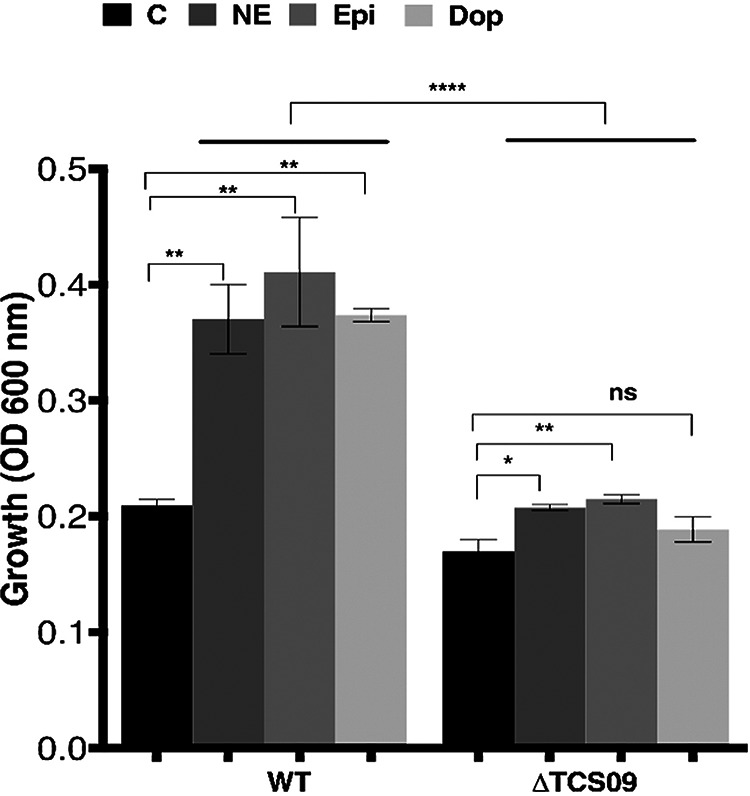
TCS09 is required for S. pneumoniae catecholamine-induced growth. The growth profiles of the wild-type (WT) and Δ*tcs09* strains are shown. The mutant and wild type were cultured for 18 h in serum-SAPI medium with no additions (control) (C) or with the addition of 50 μM norepinephrine (NE), 50 μM epinephrine (Epi), or 5 μM dopamine (Dop). The values shown are SEM from triplicate readings (*n* = 3). Significant differences were seen comparing the stress hormone response of each strain to that of its control using one-way ANOVA and Dunnett’s multiple-comparison test. *, *P* < 0.05; **, *P* < 0.01; ****, *P* < 0.0001; ns, not significant.

Having previously established the NE-induced growth enhancement of S. pneumoniae by catecholamine hormones (15), we wished to determine the genetic basis of this growth enhancement and its impact on pneumococcal translocation into the lungs. As TCS histidine kinases such as QseC and QseE have been proposed as catecholamine receptors in several Gram-negative species (5, 6), we mutated all 13 known TCSs in S. pneumoniae strain D39 and tested if the response to catecholamines was affected. Although 12 TCS mutants responded to stress hormones similarly to the wild type (wt), mutation of TCS09 was found to significantly reduce the responsiveness to all three catecholamines tested compared to the wild type (*P* < 0.0001) (Fig. 3).

**FIG 4:**
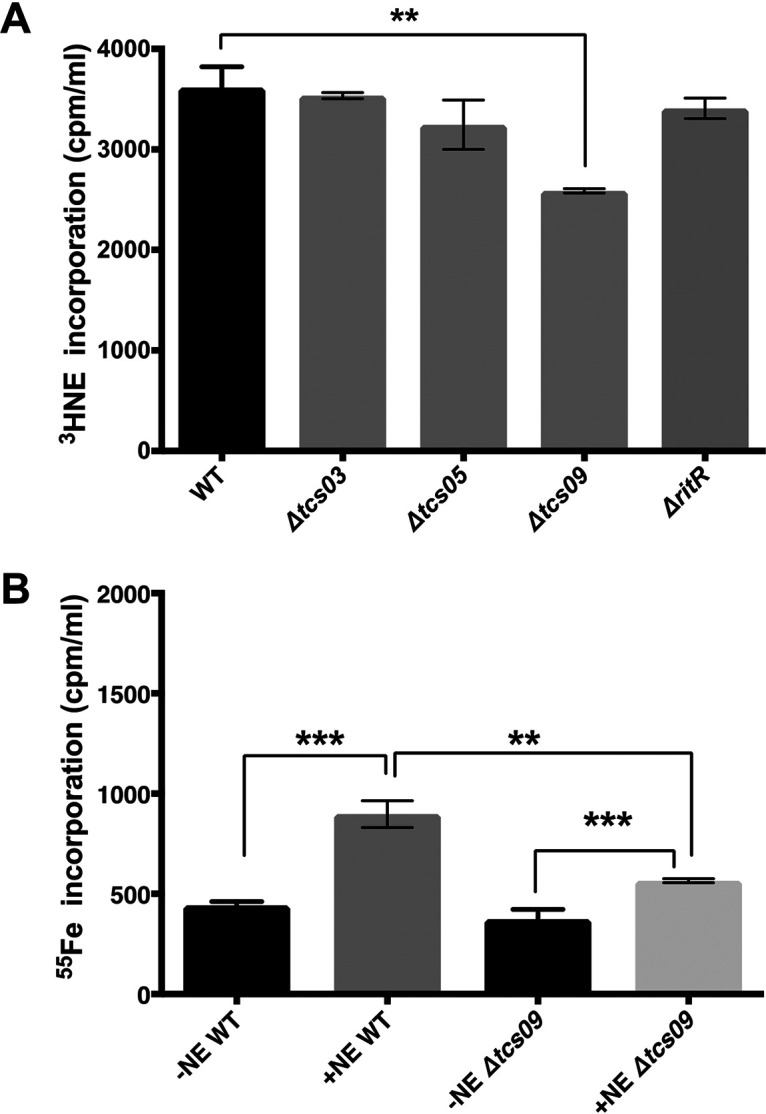
TCS09 is involved in NE-mediated iron and Tf uptake. (A) Uptake of [^3^H]NE by pneumococcal wild-type strain D39 and the Δ*tcs03*, Δ*tcs05*, Δ*tcs09*, and Δ*ritR* mutants after 24 h of incubation in serum-SAPI medium at 37°C under microaerobic conditions. (B) Uptake of iron from transferrin (in the form of ^55^Fe) in the absence (−NE) or presence (+NE) of 50 μM NE for the wild type and the Δ*tcs09* mutant (*n* = 3). The values shown are means from triplicate counts (**, *P* < 0.01; ***, *P* < 0.001).

Stress hormone promotion of microbial growth involves catecholamine uptake and the ability to access host iron stores such as transferrin (2, 15, 19). Figure 4A shows that radiolabeled NE internalization was significantly reduced in the Δ*tcs09* mutant relative to the wild type and other pneumococcal TCS mutants (*P* < 0.01). The role of TCS09 in iron acquisition mediated by the catecholamine NE (3, 4) was determined by measuring the uptake of Fe from ^55^Fe-labeled transferrin (^55^Fe-Tf) (Fig. 4B). For the S. pneumoniae wild type, the presence of NE enabled it to acquire more Fe from ^55^Fe-Tf than with NE-free cultures (*P* < 0.001); however, ^55^Fe incorporation was reduced in the Δ*tcs09* cultures compared to the NE-treated wild-type culture (*P* < 0.01). These data collectively confirm a role for TCS09 in pneumococcal catecholamine growth responsiveness, catecholamine internalization, and host iron uptake.

### TCS09 plays a role in the regulation of *cps* in the presence of NE.

**FIG 5:**
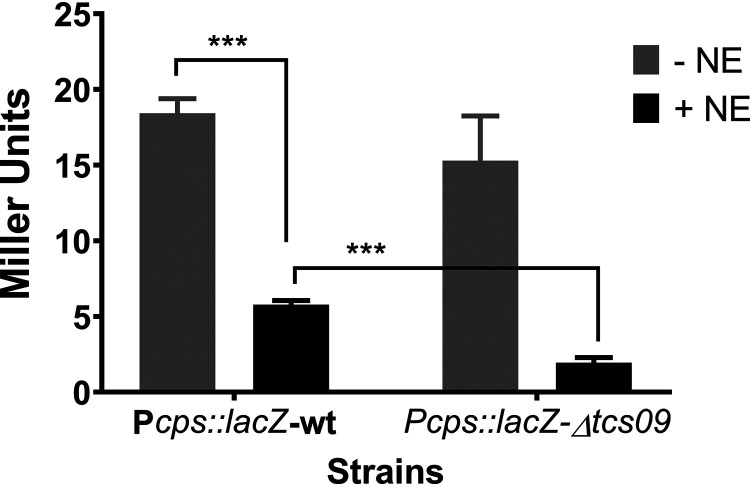
TCS09 is involved in the NE-mediated decrease in *cps* locus expression. The β-galactosidase activity levels of P*cps*::*lacZ*-wt and P*cps*::*lacZ*-Δ*tcs09* reporter strains in the presence (+) or absence (−) of 50 mM NE were determined. Activity is expressed in Miller units per 10^7^ CFU (nanomoles of *p*-nitrophenol per minute per milliliter). Error bars indicate the SEM. Values are the averages from three independent experiments, each with three replicates (***, *P* < 0.001).

We found that NE reduces capsule synthesis, as measured by glucuronic acid production, in S. pneumoniae (Fig. 2F), and this may be mediated by transcriptional regulation of the *cps* locus. A reporter strain was constructed with a chromosomally integrated copy of *lacZ* whose expression is driven by the promoter of the *cps* locus (P*cps*::*lacZ*). The effect of NE on the expression of the *cps* promoter was shown by the >3-fold decrease in β-galactosidase activity in the presence of NE (*P* < 0.001) (Fig. 5). The reduction in *cps* locus transcription is consistent with the observed decrease in glucuronic acid production upon NE treatment (Fig. 2F).

To investigate if the reduction of capsule synthesis involves the TCS09 regulatory system, β-galactosidase activity in the wild-type and Δ*tcs09* backgrounds was determined with and without NE (Fig. 5). In the presence of NE, β-galactosidase activity was significantly lower in the mutant background than in the wild type, indicating that TCS09 acts as the activator of the pneumococcal capsule locus. To determine whether TCS09 expression is directly affected by NE, we constructed a reporter strain that expressed *lacZ* under the control of the *tcs09* promoter. The expression of β-galactosidase in the presence or absence of NE in the strain containing P*tcs09*::*lacZ* did not change (*P* > 0.05) (data not shown). This suggests that the expression of TCS09 itself is not responsive to stress hormone recognition in terms of transcriptional control through the TCS09 response regulator.

A previous analysis of the TCS09 regulon by Hendriksen et al. demonstrated that it has an involvement in carbohydrate metabolism and the uptake of sugars such as mannose and fructose (20). Because the serum in the serum-SAPI medium used in this study contains glucose, mannose, and galactose (21), it was necessary to determine if the reduction of the growth response seen in the TCS09 mutant was due to a defect in sugar metabolism and not due to a loss of a catecholamine receptor. Hence, the Δ*tcs09* mutant’s growth was tested in the presence of glucose and galactose as the sole carbon sources, and the profile obtained was found to be very similar to that of the wild type, ruling out the possibility of a sugar metabolism defect (Fig. S4).

10.1128/mBio.02569-21.4 FIG S4 Growth profiles of pneumococcal strains in CDM supplemented with 55 mM galactose or glucose. Data are derived from 3 independent experiments, each in triplicates. Red line, wt; purple line, Δ*ritR*; blue line, Δ*tcs05*; green line, Δ*tcs09*. Download FIG S4, PDF file, 0.2 MB. Copyright © 2021 Alghofaili et al. 2021 Alghofaili et al. https://creativecommons.org/licenses/by/4.0/ This content is distributed under the terms of the Creative Commons Attribution 4.0 International license.

## DISCUSSION

**FIG 6:**
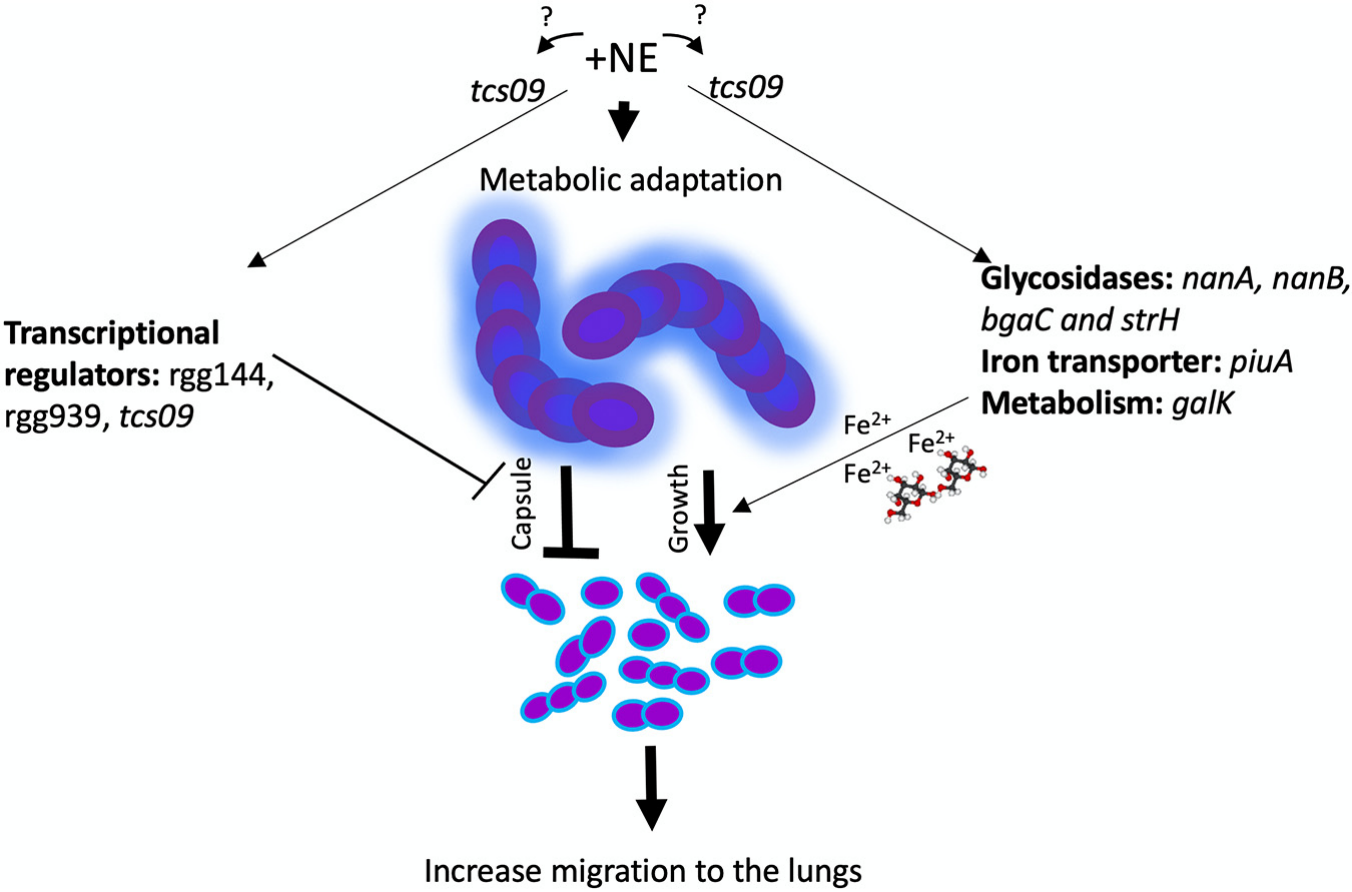
Model illustrating the potential mechanisms by which norepinephrine (NE) exerts its impact on pneumococci for increased translocation from the nasopharynx into the lungs. Block arrows indicate downregulation, while pointed arrows show upregulation. Fe^3+^ is transferrin-delivered iron, and the stick model represents the host-derived carbon source galactose. The blue outline around the pneumococcal cells represents capsule. Question marks indicate whether TCS09 is indirectly or directly involved in NE recognition.

It has been suggested that pneumococcal adaptive responses to various environmental conditions may act as a trigger for the transition from a carriage state to a disease state (22–24). Thus, we hypothesized that factors such as stress hormones may be important for the transition from carriage to infection. Our data show that the pneumococcus preexposed to host stress signals is more able to translocate from the nasopharynx into the lungs than cultures grown in the absence of hormone. The observed impact of NE on pneumococcal translocation from the nasopharynx to the lungs is very likely a multifactorial process involving effects on pneumococcal cell size, capsule production, and metabolic reprogramming (Fig. 6). Our data were reproducible with two reference strains, D39 and TIGR4. However, the generalizability of the findings to other pneumococcal strains is unknown until they are validated with other serotypes.

NE treatment increases the growth rate and yield, possibly by the upregulation of glycosidases, which are important for the release of host glycans, and by the acquisition of iron from iron-containing proteins (Fig. 4B) (3, 4). The increased availability of iron is also consistent with the elevated expression of an iron transporter as evidenced by quantitative real-time PCR (qRT-PCR) (Table 1). Morphological analysis of S. pneumoniae using light microscopy showed that the cells exposed to NE appeared as diplococci, while cells of the control group were seen as short chains. Interestingly, this agrees with the observation that pneumococci from clinical samples typically exist in a diplococcal form (25), while a short-chain phenotype is more typical for asymptomatic colonization, particularly within complex multicellular biofilm communities (26, 27). This study identified a significant reduction of pneumococcal cell size after NE treatment as shown in Fig. 2C to E. Pneumococcal cells are normally between 0.5 and 1 μm in diameter (28), and a reduction in cell size has multiple implications for the microbial phenotype. For example, smaller cells would have a higher surface-to-volume ratio, promoting nutrient exchange, which would enhance host proliferation. This is consistent with our previous data, also repeated in this study, that showed that the addition of catecholamines enhances the pneumococcal growth rate and yield (15).

NE treatment of the pneumococcus decreases the transcription of the capsule locus and its synthesis. A decrease in capsule synthesis is the likely reason for the reduction in cell size and the elevated growth rate. Capsule is essential for pneumococcal virulence; therefore, our two observations linking NE treatment with increased dissemination into the lungs and reduced capsule synthesis may initially look contradictory. However, it should be remembered that increased capsule production can also decrease attachment to epithelial cells. Analysis of serotypes with higher carriage prevalences has shown such strains produce thicker capsules than low-carriage-prevalence serotypes (29). Hammerschmidt et al. demonstrated that a reduction in capsular material led to up to a 10^5^-fold enhancement in the pneumococcal capacity to adhere to and invade epithelial cells (30). NE, by reducing capsule synthesis and stimulating the growth rate and yield, leads to a phenotype that potentiates translocation from the nasopharynx into the lungs (Fig. 1).

To determine the timing of NE induction of pneumococcal phenotypic changes, we exposed pneumococci to NE for short and long periods. Short and long exposures to NE are relevant *in vivo* as high catecholamine levels, which are regulated by multiple mechanisms, may be only short term (minutes) in periods of stress or over extended periods of time if administered therapeutically to reduce airway inflammation in intubated patients (31). In this study, we observed that long exposure to NE led to phenotypic changes, suggesting that the basis of these observed changes was metabolic reprogramming.

NE pretreatment of pneumococci allowed the deduction of whether the observed phenotypic changes were due to metabolic reprogramming. Metabolomic analysis indicated that NE-treated S. pneumoniae had lower intracellular levels of lactic acid, phosphoenolpyruvate, and fumarate. It is likely that reduced phosphoenolpyruvate is due to increased consumption of this metabolite for both energy generation and the phosphoenolpyruvate-dependent sugar phosphotransferase system, which is consistent with the increased growth rate and yield observed in the presence of NE. Lactic acid is the main fermentative end product of S. pneumoniae when the preferred carbon source, glucose, is used. However, the growth medium used in this study contained only 2 mM glucose, although serum is known to be rich in N-linked glycans, such as transferrin, which is rich in mannose. Hence, due to the use of other carbon sources in the media, a decrease in the lactic acid level is not unexpected. Fumarate can be produced either through the Embden-Meyerhof-Parnas pathway by pyruvate reductive carboxylation or through the Krebs cycle where the interaction of oxalacetate with acetyl-CoA leads to the formation of citric acid, which subsequently undergoes two decarboxylation steps and other transformations to generate oxaloacetates, one of which is fumaric acid (32). The Krebs cycle is the main source of fumaric acid, but the pneumococcus does not have all the enzymes of the full Krebs cycle. Therefore, it is likely that fumarate is generated through fermentation. Currently, we do not know how fumarate potentiates pneumococcal translocation to the lungs after NE treatment, but in enterohemorrhagic Escherichia coli, fumarate was reported to regulate virulence (33). Our results clearly show that NE treatment induces significant metabolic alterations in S. pneumoniae, but further work is needed to reveal the full extent of these metabolic changes and by which mechanisms they lead to phenotypic alterations.

In this study, we have explored the genetic basis of stress hormone recognition and processing. TCSs enable bacteria to sense and react to environmental changes within their host, and their possible role in stress hormone-bacterium interactions has been identified in some Gram-negative bacteria (34). To determine their role in pneumococcal stress hormone signal recognition and processing, in this study, 13 TCSs were mutated, and the mutants were analyzed for their response to the catecholamines norepinephrine, epinephrine, and dopamine. The results showed that only the TCS09 mutant most significantly lost its ability to respond to the 3 hormones, which is for the first time a direct demonstration of the catecholamine responsiveness of a TCS in Gram-positive bacteria. Additionally, the multiple loss of adrenergic and dopaminergic catecholamine recognition when TCS09 was removed indicates that receptors for individual catecholamines do not exist within the pneumococcus. This is in marked contrast to the specificity of catecholamine recognition by alpha, beta, and dopaminergic receptors within eukaryotic species (35). TCS09 also plays a number of other roles in S. pneumoniae physiology. Recently, Hirschmann et al. demonstrated the importance of TCS09 for pneumococcal virulence, metabolic fitness, and resistance against oxidative stress through its control of carbohydrate metabolism, cell wall integrity, and capsular polysaccharide synthesis (36). Its target loci include the genes important for competence, *agaR*, which is also known as CpsR and is involved in capsule locus expression, and the *aga* operon, which is involved in galactose metabolism (37). In addition, *bgaC*, *agaS*, and *galM*, all involved in galactose metabolism, manifested increased mRNA levels in TCS09 mutants. The results of this study also show the importance of TCS09 in the regulation of galactose metabolism and capsule synthesis when pneumococci are exposed to NE. It was found that NE-mediated downregulation of *cps* expression by TCS09 was indirect as NE did not have any effect on the induction of *tcs09*. This shows either that there are other regulatory or effector proteins involved in TCS09-mediated *cps* expression or that NE treatment may manifest its impact through the phosphorylation of TCS09. Previously, Gonzales et al. suggested a role for pneumococcal TSC03 and TSC06 in promoting pneumococcal growth in the presence of norepinephrine (38). However, the findings of this study by mutant analyses and reporter assays could not link these TCSs to pneumococcal catecholamine biology.

In our study, the loss of TCS09 also caused a reduction in the uptake of radiolabeled NE and ^55^Fe from ^55^Fe-labeled Tf (Fig. 4), which indicates a role in catecholamine-mediated growth induction for TCS09 and suggests against a sole metabolism role. While mutation of TCS09 led to a reduction of [^3^H]NE uptake, it did not totally abolish it. This suggests that the pneumococcus may have other receptor pathways involved in the pneumococcal response to NE. In Gram-negative species, a functional siderophore system (e.g., enterobactin synthesis [EntA] and TonB) is needed for catecholamine growth induction (2–4). It has been shown that catecholamines can form a pseudo-siderophore-like chemical complex when ferric iron is present that has some similarity to siderophore-mediated acquisition. Gram-positive bacteria can use catecholamines to acquire iron from host iron-binding proteins such as transferrin and lactoferrin, but the mechanistic role of siderophores or other secreted iron-related effectors is uncertain (39). It is possible that in Gram-positive species such as the pneumococcus, catecholamines can be used directly as a kind of siderophore, and we show here that both transferrin-derived iron and the catecholamine NE are directly internalized into the pneumococcus (Fig. 4A and B).

In conclusion, our data clearly demonstrate that pneumococci exposed to catecholamines potentiate their ability to translocate from the nasopharynx into the lungs. Exposure to catecholamines can be due to the endogenous production of these neurochemicals or by the exogenous administration of these compounds as inotropic agents for the clinical management of acutely ill patients with heart and kidney dysfunction. These individuals are at an increased risk of acquiring invasive pneumococcal infections, and additional side effects of catecholamine medications prescribed to them should be taken into account as a potential infection risk factor. Collectively, the evidence in this study suggests that the pneumococcus has mechanisms to recognize and process host stress hormones to augment its transition from colonization to translocation into the lungs. This transition is mediated by stress hormone-induced changes in capsule synthesis and cell size and is controlled by TCS09. It is therefore important that future studies should focus on a detailed characterization of the TCS09 regulon in pneumococcal stress hormone recognition and processing.

## MATERIALS AND METHODS

### Bacterial strains, plasmids, reagents, and growth conditions.

The bacterial strains and plasmids used in this study are shown in Table S1 in the supplemental material. Human serum transferrin, ferric nitrate, and the catecholamine hormones were purchased from Sigma Chemical Co. (Poole, Dorset, UK). The radioisotopes ^55^FeCl_3_ (IES) (specific activity, 5 mCi/mg Fe) and ^3^H[NE] (TRK584,l-[7,8-^3^H]norepinephrine) were obtained from Amersham Life Sciences, UK.

10.1128/mBio.02569-21.5 TABLE S1 Bacterial strains and plasmids used in this study. Download Table S1, PDF file, 0.2 MB. Copyright © 2021 Alghofaili et al. 2021 Alghofaili et al. https://creativecommons.org/licenses/by/4.0/ This content is distributed under the terms of the Creative Commons Attribution 4.0 International license.

Streptococcus pneumoniae D39 type 2 and its isogenic mutants were routinely grown under microaerobic conditions at 37°C in brain heart infusion (BHI) broth or on plates of blood agar base (Oxoid) containing 5% (vol/vol) horse blood. When needed, both growth media were supplemented with 100 μg/ml of spectinomycin and/or 15 μg/ml of tetracycline. A serum-based minimal medium (serum-SAPI) (39) supplemented with vitamins of Sicard’s medium (40) was also used in this study and, when required, with additions of NE, Epi, Dop, or ferric nitrate, each at 50 μM. The pneumococcal strains were inoculated into serum-SAPI medium with a low inoculum size of 10^2^ to 10^3^ CFU/ml (19, 41).

Growth time course experiments were carried out using a Multiskan Go microplate spectrophotometer (Thermo Scientific, UK). For growth assays, 100-μl replicates of serum-SAPI medium or chemically defined medium (CDM) supplemented with or without the stress hormones, Fe, and selective amino acids as well as bacterial suspensions of around 10^2^ to 10^3^ CFU/ml were added to wells of a flat-bottom 96-well microtiter plate. This mixture was then incubated for 20 h at 37°C with hourly readings of the optical density at 600 nm (OD_600_). The maximum growth yield was considered to be the highest OD_600_ value reached by the bacterial culture, whereas the specific growth rate was calculated by linear regressions of growth plots of ln(OD_600_) against time according to previously described protocols (42).

### Mutant and reporter strain construction.

To determine the role of two-component regulatory systems (TCSs) in catecholamine sensing and processing, all 13 TCSs within the S. pneumoniae genome were mutated by allelic replacement mutagenesis using a splicing by overlap extension (SOEing) protocol (22, 24). The primers used for mutations are shown in Table S2.

10.1128/mBio.02569-21.6 TABLE S2 Oligonucleotide primers used in this study. The LF/F and RF/R primers were designed to amplify the target gene (wild type) or the Spc^r^ gene (mutants) along with 800 bp of the left and 800 bp of the right flanks (up- and downstream of the mutated region). The LF/F-X and Spe/R primers were designed to interrogate the mutated region by amplifying the left flank of the targeted gene and the inserted Spc^r^ cassette, while Spe/F and RF/R amplified the cassette and the right flanking regions of the targeted gene. The Spc^r^ cassette (1,158 bp) was also amplified using the Spe/F and Spe/R primers. Fusion primers were used for the construction of *lacZ* fusions. Underlined nucleotides refer to the incorporated restriction enzyme recognition sites. “RTF” or “RTR” indicates the primer sets used for real-time quantitative PCR. Download Table S2, PDF file, 0.03 MB. Copyright © 2021 Alghofaili et al. 2021 Alghofaili et al. https://creativecommons.org/licenses/by/4.0/ This content is distributed under the terms of the Creative Commons Attribution 4.0 International license.

Chromosomal transcriptional *lacZ* fusions to the target promoters were constructed via double crossover in the *bgaA* gene by using the integrative plasmid pPP2 (43). Upon integration, *bgaA* is deleted, which reduces the background β-galactosidase activity. The putative promoter regions were amplified after their assessment using BPROM (44). The amplicons were digested with SphI and BamHI and ligated into similarly digested pPP2. An aliquot of the recombinant plasmid was transformed into S. pneumoniae as previously described (22). All plasmid constructs were confirmed by sequencing.

β-Galactosidase reporter activity assays were performed according to previously published protocols (22, 43). The S. pneumoniae promoter responsiveness to stress hormones was determined by measuring β-galactosidase activity, encoded by a promoterless *lacZ* gene under the transcriptional control of the promoter under investigation.

### Capsule extraction and glucuronic acid assay.

Pneumococcal polysaccharide capsule was extracted as previously described (22). The bacterial culture was treated with 1% Zwittergent 3-14 detergent in 100 mM citric acid (pH 2.0). This mixture was then incubated at 50°C for 30 min and centrifuged at 10,000 rpm before transferring the supernatant into a new tube, when absolute ethanol was added to a final concentration of 80%. Next, the solution was placed at 4°C for 20 min followed by recentrifugation, and the resultant pellet was dissolved in 200 μl of distilled water. Glucuronic acid measurement was carried out according to a protocol described previously by Bitter and Muir (45). The protocol used a 0.025 M sodium tetraborate solution, which was added to the capsule samples, which were then incubated at 100°C for 10 min and cooled in an ice bath before adding a 0.125% (wt/vol) carbazole solution (45). The samples were subsequently heated at 100°C for 10 min and cooled to room temperature. Finally, the absorbance of the glucuronic acid released was measured at 530 nm using a spectrophotometer.

### Norepinephrine and transferrin uptake assays.

To test the ability of pneumococcal strains to acquire iron from transferrin (Tf), serum medium containing filter-sterilized ^55^Fe-Tf (2 × 10^5^ cpm ml^−1^) was supplemented with 50 μM norepinephrine. Washed pneumococcal cultures were added at 10^7^ CFU/ml to ensure that the growth levels of catecholamine-treated and control cultures were the same before being incubated for 24 h at 37°C in a humidified 5% CO_2_ incubator. For analysis of catecholamine internalization, pneumococcal wild-type and selected mutant cultures were similarly grown but supplemented with 1 × 10^5^ cpm/ml of [^3^H]norepinephrine. For these assays, pneumococcal cultures grown overnight were harvested by centrifugation at 10,000 × *g* for 10 min, washed in phosphate-buffered saline (PBS), and assayed for cell numbers and for radiolabel incorporation as described above for the ^55^Fe-Tf assays. Internalization of [^3^H]norepinephrine was measured by mixing washed S. pneumoniae cells with 2 ml of emulsifier-safe scintillant (Canberra-Packard, Pangbourne, UK) for counting in the tritium channel of a Minaxi Tri-Carb 400 series scintillation counter (Canberra-Packard).

### Metabolome analysis.

Pneumococcal extracellular and intracellular metabolites were prepared according to a previously described protocol (46). S. pneumoniae D39 was cultured overnight in serum-SAPI medium with no additions or in the presence of 50 μM NE. The pneumococci were treated with 10 mM sodium azide and sedimented at 8,000 × *g* at 5°C for 15 min. They were then washed with 40 mM Tris-HCl (pH 7.4) before extraction with cold methanol containing 0.2 mM norvaline as an internal standard. The samples, including a serum-SAPI medium control, as well as extracellular and intracellular bacterial metabolites were sent to MS-Omics (Frederiksberg, Denmark) for analysis. Samples were derivatized with methyl chloroformate using a modified version of the protocol described previously by Smart et al. (47). All samples were analyzed in a randomized order. Analysis was performed using a gas chromatography (GC) system (catalog no. 7890B; Agilent) coupled with a quadrupole detector (catalog no. 59977B; Agilent). The system was controlled by ChemStation (Agilent). Raw data were converted to netCDF format using ChemStation (Agilent), before the data were imported and processed in Matlab R2014b (Mathworks, Inc.) using PARADISe software (48). In terms of metabolite identity assignment, derivatization with methyl chloroformate, especially carboxy-metabolites, can be measured, such as amino acids, most tricarboxylic acid (TCA) cycle metabolites, and free fatty acids. After data processing with PARADISe software, the metabolites are either identified using authentic chemical standards, which are compared to retention times and mass spectra, or annotated based on library matching of the acquired MS spectra with the NIST library. The annotation might be incorrect; however, the compound will most likely be of a similar structure. If there is no hit with the NIST library, the compounds are labeled as unknowns.

### *In vivo* infection experiments.

The pneumococcal inoculum was prepared in two ways. The first inoculum was prepared as described previously except that pneumococci were exposed to 50 μM NE for 30 min before inoculation into mice (49). The second inoculum was prepared from a pneumococcal culture grown in serum-SAPI medium supplemented with 50 μM NE as previously described (15). The control cultures for both methods were not exposed to NE. The stocks were kept frozen at −80°C until needed.

For infection studies, female CD1 outbred mice, 8 to 10 weeks old, were used (Charles River). For mouse inoculation experiments, we used conditions that allow stable intranasal colonization, which is achieved by reducing the inoculum size and dose (50). Each mouse received 5 × 10^5^ CFU in 10 μl PBS intranasally under light anesthesia using 2.5% (vol/vol) isoflurane (Isocare) over oxygen (1.4 to 1.6 liters/min). The determination of the precise inoculum size was done by viable counting after dose administration. During the course of infection (7 days), the animals were monitored daily for disease signs such as lethargy or hunched or piloerect postures. Following intranasal inoculation at days 0, 2, and 7, nasopharyngeal wash fluid, BAL fluid, and lung tissues were collected from each group of mice. The bacterial counts in these samples were determined by inoculating the serially diluted samples onto blood agar plates containing 5 μg/ml of gentamicin to prevent contamination.

### Gene expression analysis in pneumococci recovered from the nasopharynx.

To determine whether NE-treated bacteria retain the *in vitro*-observed physiological changes (capsular and metabolomic) during colonization, we determined the expression of selected genes in pneumococci recovered from the nasopharynx. Two groups of mice were infected with pneumococci grown in either the absence or presence of 50 μM NE as described above; at 7 days postinfection, mice were killed by cervical dislocation; the nasopharyngeal lavage fluid was obtained by using 500 μl TRIzol reagent; and the extraction of RNA was done as recommended by the manufacturer (Thermo Fisher, Loughborough, UK). Before use, the RNA was treated with Turbo DNase (Thermo Fisher). First-strand cDNA synthesis was performed on approximately 400 ng of DNase-treated total RNA using 200 U of SuperScript II reverse transcriptase (Thermo Fisher) at 42°C for 55 min and random hexamers. cDNA (10 ng) was amplified in a 20-μl reaction volume that contained 1× SYBR green PCR master mix and 3 pmol of each primer (Table S2). The transcription levels of specific genes were normalized to the transcription level of *gyrB*, which was amplified in parallel with primers SPD0709F and SP0709R. The results were analyzed by the comparative threshold cycle (*C_T_*) method (51).

### Ethics statement.

Mouse experiments were performed under project (permit no. 60/4327) and personal (permit no. 80/10279) licenses according to United Kingdom Home Office guidelines under the Animals Scientific Procedures Act 1986 and University of Leicester ethics committee approval. The protocol used was approved by both the UK Home Office and the University of Leicester ethics committee. Where indicated, the procedures were carried out under anesthesia with isoflurane. Animals were housed in individually ventilated cages in a controlled environment and frequently monitored after infection to minimize suffering.

### Statistical analysis.

All statistical analyses were performed via GraphPad Prism software 6.0 (GraphPad, CA, USA), and all experiments were performed in triplicate on at least 3 separate occasions, unless stated otherwise. Data are expressed as means ± standard errors of the means (SEM). Where appropriate, statistical analysis was first performed using an unpaired *t* test or one-way analysis of variance (ANOVA) followed by Dunnett’s multiple-comparison tests. Significance is defined by asterisks in the figures (*, *P* < 0.05; **, *P* < 0.01; ***, *P* < 0.001; ****, *P* < 0.0001).
