# Correction for Alghofaili et al., “Host Stress Signals Stimulate Pneumococcal Transition from Colonization to Dissemination into the Lungs”

**DOI:** 10.1128/mbio.02872-24

**Published:** 2024-10-28

**Authors:** Fayez Alghofaili, Hastyar Najmuldeen, Banaz O. Kareem, Bushra Shlla, Vitor E. Fernandes, Morten Danielsen, Julian M. Ketley, Primrose Freestone, Hasan Yesilkaya

**Affiliations:** 1 Department of Medical Laboratory Sciences, College of Applied Medical Sciences, Majmaah University, Majmaah, Saudi Arabia; 2 Department of Respiratory Sciences, University of Leicester, Leicester, United Kingdom; 3 Department of Biology, College of Science, University of Sulaimani, Sulaymaniyah, Iraq; 4 Department of Biology, College of Science, University of Mosul, Mosul, Iraq; 5 MS-Omics ApS, Frederiksberg, Denmark; 6 Department of Genetics and Genome Biology, University of Leicester, Leicester, United Kingdom

## AUTHOR CORRECTION

Volume 12, no. 6, e02569-21, 2021, https://doi.org/10.1128/mBio.02569-21. Page 1: The affiliations and affiliation numbers should appear as shown in this correction.

Page 13, Acknowledgments, paragraph 1: The following should be added. “F.A. extends appreciation to the Deanship of Postgraduate Studies and Scientific Research at Majmaah University for funding this research work through project number R-2024-1314.”

