# Supplementary figures and images for: Host Stress Signals Stimulate Pneumococcal Transition from Colonization to Dissemination into the Lungs
Source: mBio. 2021 Oct 26;12(6):e02569-21. doi: 10.1128/mBio.02569-21 (PMC8546540; doi:10.1128/mBio.02569-21)

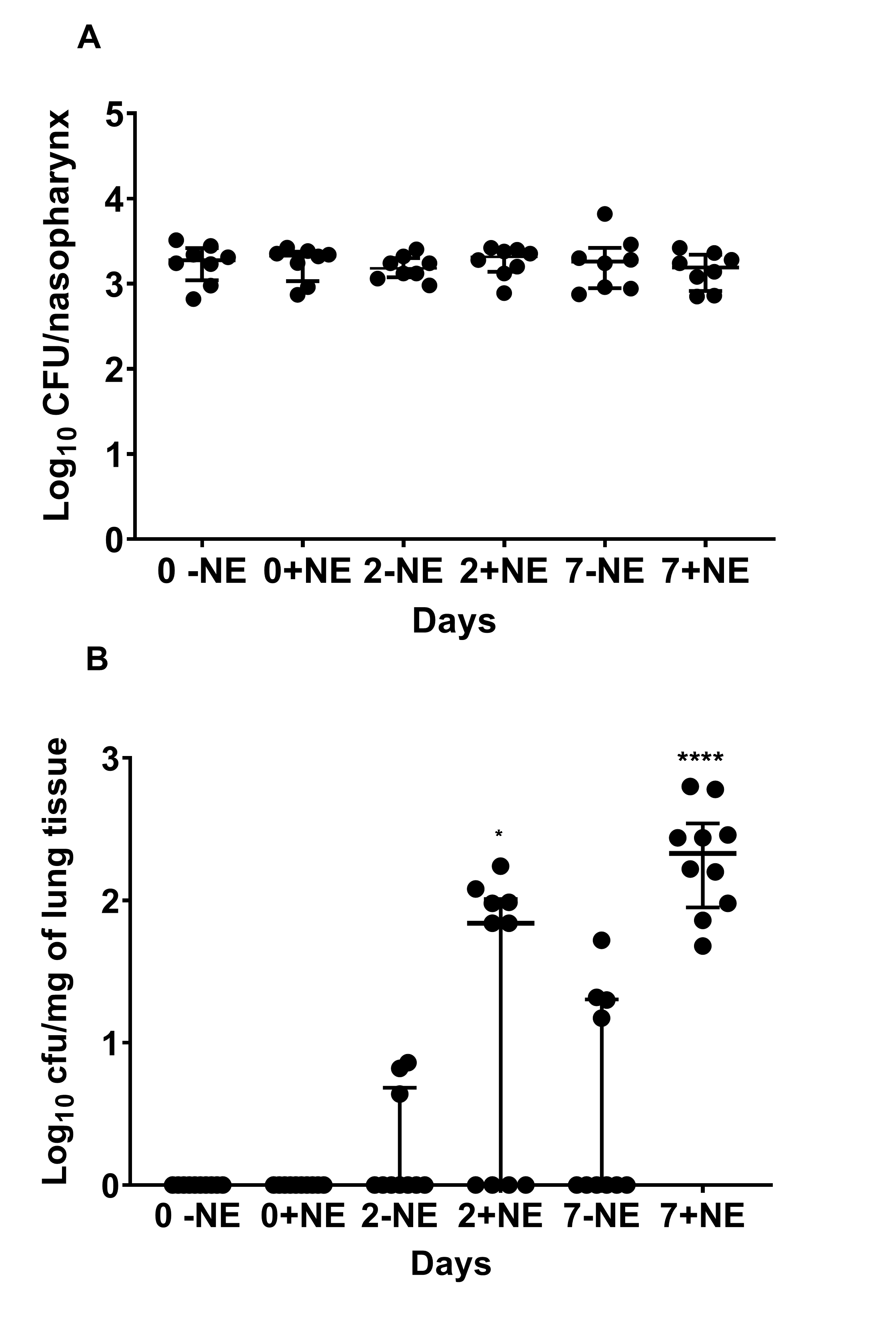

Supplement: FIG S1 [file mbio.02569-21-sf001.tif]

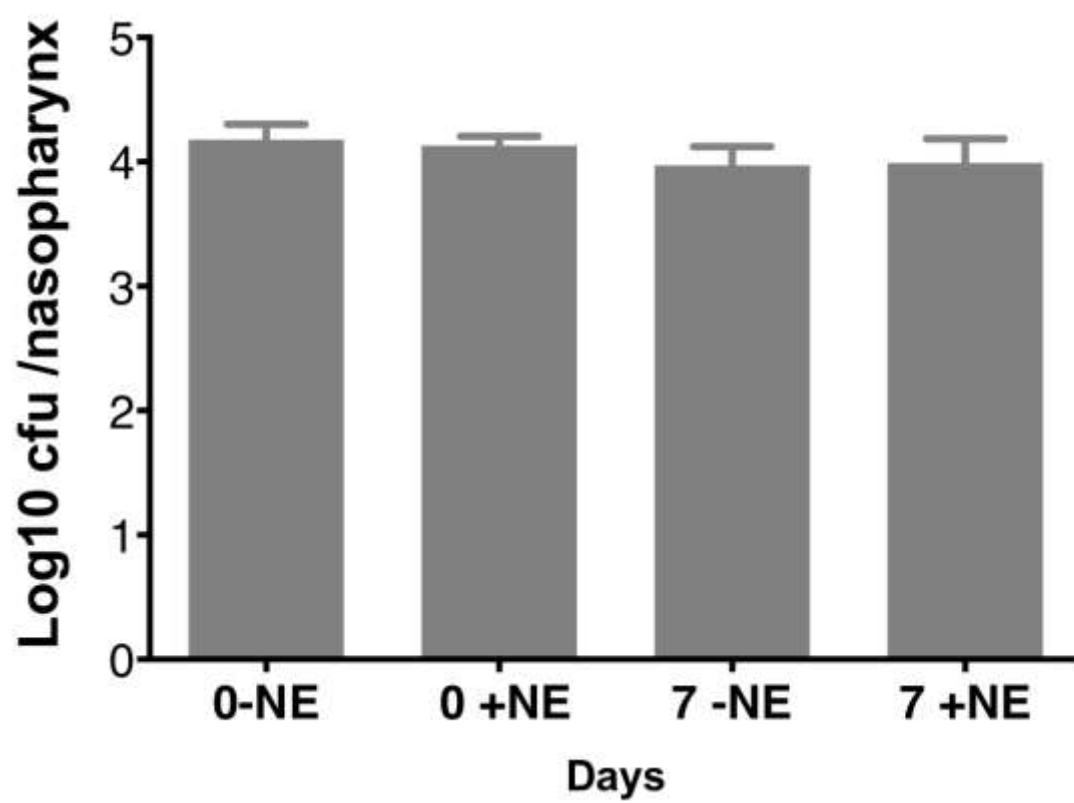

Supplement: FIG S2 [file mbio.02569-21-sf002.pdf]

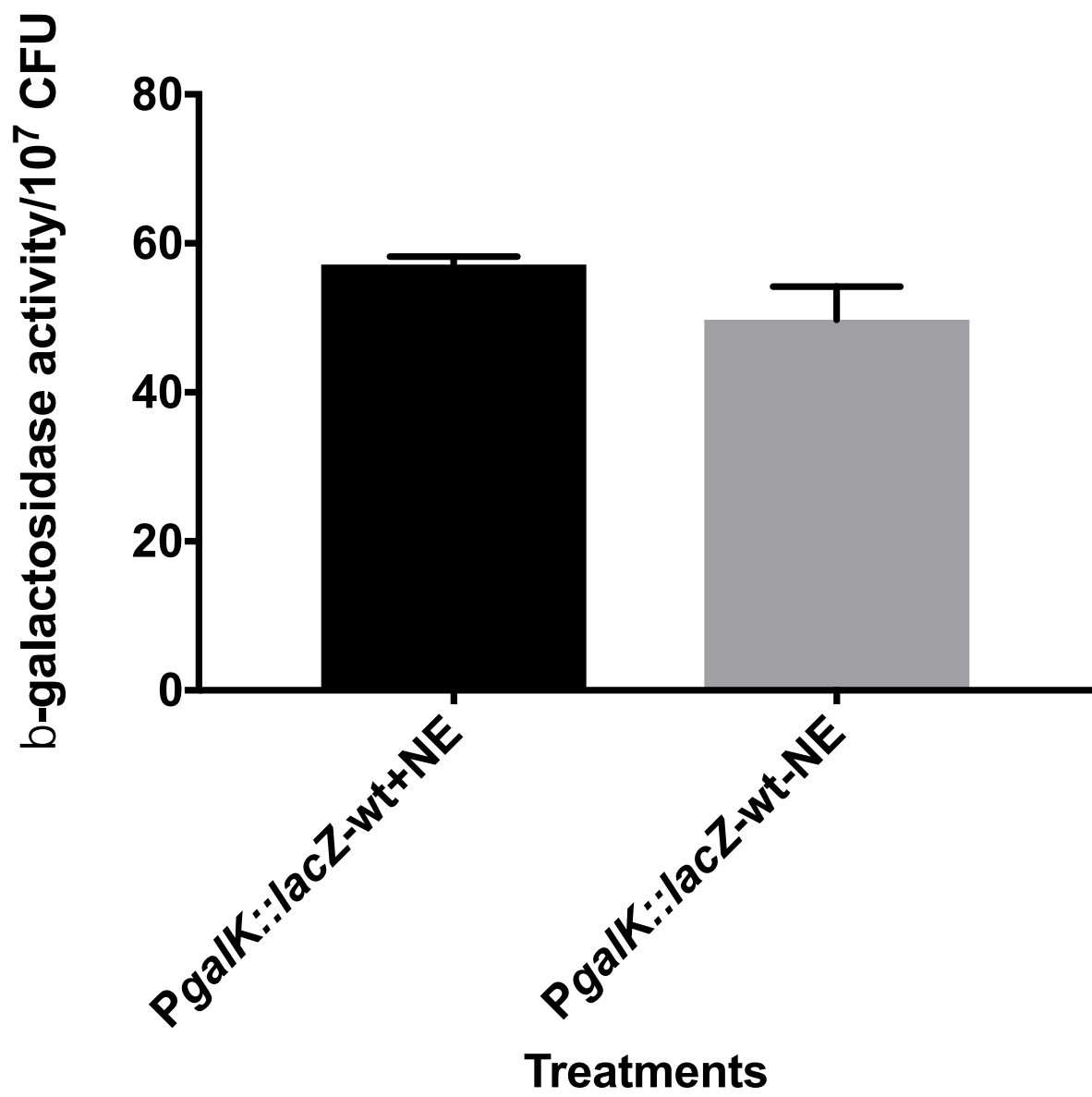

Supplement: FIG S3 [file mbio.02569-21-sf003.pdf]

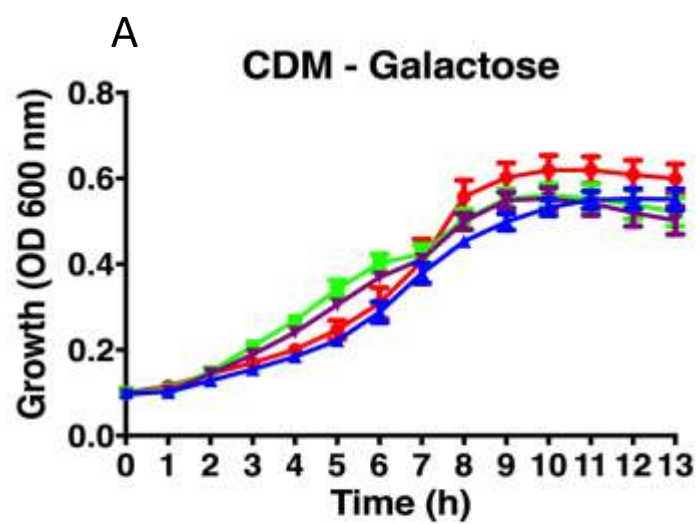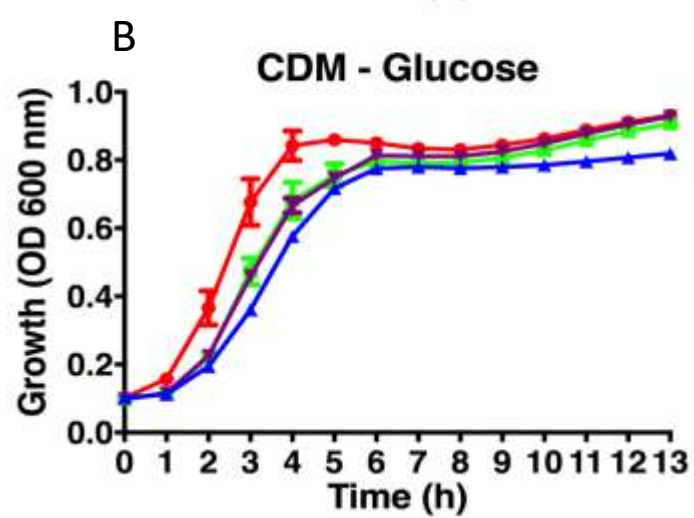

Supplement: FIG S4 [file mbio.02569-21-sf004.pdf]
