# Supplementary material for: Host Stress Signals Stimulate Pneumococcal Transition from Colonization to Dissemination into the Lungs
Source: mBio. 2021 Oct 26;12(6):e02569-21. doi: 10.1128/mBio.02569-21 (PMC8546540; doi:10.1128/mBio.02569-21)
Supplement: TABLE S1 [file mbio.02569-21-st001.pdf]

**Strains/Plasmids**	**Description/Use**	**Source**
D39	Serotype 2 strain	Laboratory stock
Δ*tcs012*	Spc^R; ΔSPD_2063	This study
Δ*tcs013*	Spc^R; ΔSPD_0468	This study
Δ*ciaR*, (Δ*tcs05)*	Spc^R; ΔSPD_0701	This study
Δ *tcs08*	Spc^R; ΔSPD_0081	This study
Δ *tcs04*	Spc^R; ΔSPD_1908	This study
Δ *tcs03*	Spc^R; ΔSPD_0352	This study
Δ *tcs07*	Spc^R; ΔSPD_0158	This study
Δ *tcs014*	Spc^R; ΔSPD_0344	This study
Δ *tcs06*	Spc^R; ΔSPD_2020	This study
Δ *tcs010*	Spc^R; ΔSPD_0524	This study
Δ *tcs01*	Spc^R; ΔSPD_1446	This study
Δ *tcs09*	Spc^R; ΔSPD_0574	This study
Δ *tcs011*	Spc^R; ΔSPD_1798	This study
ΔpPP2-D39wt	Tet^R; ΔpPP2-D39-wt	This study
P*ciaR* -*lacZ*-wt	Tet^R; P*ciaR*-*lacZ*-wt	This study
P*ciaR*-*lacZ*-Δ*ciaR*	Spc^R, Tet^R; *ciaR*-*lacZ*-Δ*ciaR*	This study
P*tcs*03-*lacZ*-wt	Tet^R; *Ptcs03*-*lacZ*-wt	This study
P*tcs*03-*lacZ*-*Δtcs03*	Spc^R, Tet^R; Δ*Ptcs*03-*lacZ*-Δ*tcs*03	This study
Ptcs09::lacZ-wt	Tet^R;P*tcs09*-*lacZ*-wt	This study
Pcps-*lacZ*-wt	Tet^R; *Pcps*-*lacZ*-wt	Reference[1]
Pcps-*lacZ*-*Δtcs09*	Spc^R, Tet^R; *Pcps*-*lacZ*-*Δtcs09*	This study
P*galK::lacZ*-wt	Tet^R, P*galK::lacZ*-wt	This study
E.coli top10	plasmid propagation	Laboratory stock
E.coli top10	Competent cells	This study
E.coli top10	Amp^R; ΔpPP2-top10	This study
E.coli top10	Kan^R; ΔpCEP-top10	This study
**Plasmids**		
pDL278	Amplification of Spc^R (*aadA*)	Reference[2]
pPP2	Promoterless *lacZ* for transcriptional fusions; Amp^R Tet^R	Reference[3]
pCEP	Genetic complementation; Kan^R	Reference[4]
