# Supplementary material for: Host Stress Signals Stimulate Pneumococcal Transition from Colonization to Dissemination into the Lungs
Source: mBio. 2021 Oct 26;12(6):e02569-21. doi: 10.1128/mBio.02569-21 (PMC8546540; doi:10.1128/mBio.02569-21)
Supplement: TABLE S2 [file mbio.02569-21-st002.pdf]

**PRIMERS**	**5`→ 3`**
Spe/F	ATCGATTTTCGTTCGTGAAT
Spe/R	GTTATGCAAGGGTTTATTGT
LF/F- TSC12	TCAGATATGGTAAGTACGAT
LF/R- TSC12	TATTCACGAACGAAAATCGATAAACTTTCATTCAAATTCC
RF/F- TSC12	AACAATAAACCCTTGCATAACTCAAAAGTGATTGACAATTAGC
RF/R- TSC12	TTACAAGGAGGAAATATGCAAG
LF/F- TSC13	TTCTAGTTCAATCAAGAATCGA
LF/R- TSC13	TATTCACGAACGAAAATCGATATATTCTCATCTTCTTACTC
RF/F- TSC13	AACAATAAACCCTTGCATAACCTTACACTGAGCTAGGAGAG
RF/R- TSC13	AGCTGGTTAAGAGGTTAGTGTAG
LF/F- TSC05	ATGAAGGAACGGATGCTGAAAC
LF/R- TSC05	TATTCACGAACGAAAATCGATCTCTCTGCATTTTACATGAG
RF/F- TSC05	AACAATAAACCCTTGCATAACGATGTTCAGTAAACTTAAAAAAAC
RF/R- TSC05	CGCATATTTCGGACTTCTTCCA
LF/F- TSC08	AGGTGATGGTATGTTGACTCG
LF/R- TSC08	TATTCACGAACGAAAATCGATGTCTTTCCCATCTGTCTCTCC
RF/F- TSC08	AACAATAAACCCTTGCATAACGACAAACATGAAACTAAAAAG
RF/R- TSC08	GTCCTTCTCCAGCTTAGCCACC
LF/F- TSC04	GACTTGTGACAATCGCTTGAG
LF/R- TSC04	TATTCACGAACGAAAATCGATGTTTTGTCATCTATTATCTCC
RF/F- TSC04	AACAATAAACCCTTGCATAACGGAGTTATAGATGAAACGCT
RF/R- TSC04	GGTTTCAGCAAAGCCCTTAA
LF/F- TSC03	CTCAAGCGTTTATTGGCAGG
LF/R- TSC03	TATTCACGAACGAAAATCGATGTAAAATTTTCATCTTTACTCC
RF/F- TSC03	AACAATAAACCCTTGCATAACGGAGTTTTAGATGAGTTTAGC
RF/R- TSC03	GCTAGAGGTACTTGCTTGCT
LF/F- TSC07	CGGATTGACATTTCCGAGAAAG
LF/R- TSC07	TATTCACGAACGAAAATCGATACTTTATACATTTTCTCCCT
RF/F- TSC07	AACAATAAACCCTTGCATAACCTATACTATAAGATTTGTATTCC
RF/R- TSC07	CCTTGACCAAATCCTTGATATGAA
LF/F- RitR	GGCTGAAATCTTTACTGAGTGG
LF/R- RitR	TATTCACGAACGAAAATCGATGTTTCCCCATGGCTGACCTA
RF/F- RitR	AACAATAAACCCTTGCATAACGCAAGAATAGAAAAGCAGTT
RF/R- RitR	GAGCAGGTTTGCTTCTATCAAT
LF/F- TSC06	GATATGACCCAGAGATGGGAG
LF/R- TSC06	TATTCACGAACGAAAATCGATAAATGTTCATCTCTCTCCCT
RF/F- TSC06	AACAATAAACCCTTGCATAACTAGCTTATGATAAAAAATCC
RF/R- TSC06	GGATAATTCTAAGACTGGCTAAAGG
LF/F- TSC10	GAGCTTCTCAGTTCTCCTCC
LF/R- TSC10	TATTCACGAACGAAAATCGATAATTTTCATACTTTAACTGC
RF/F- TSC10	AACAATAAACCCTTGCATAACGCGAAAATGAAACGAACAGG
RF/R- TSC10	GACCTTCATCTTCTCCAGCT

LF/F- TSC01	TATCCTTTGATAACCCGCAGTC
LF/R- TSC01	TATTCACGAACGAAAATCGATTCTTGTGCATGCGCTTCTCC
RF/F- TSC01	AACAATAAACCCTTGCATAACGCATGCTTGATTGGAAACAA
RF/R- TSC01	CCACGTTCAAATACTCGGAG
LF/F- TSC09	GCCTATCCTGTCATTGATGC
LF/R- TSC09	TATTCACGAACGAAAATCGATTGTAGGTCATGCTCTGCTCC
RF/F- TSC09	AACAATAAACCCTTGCATAACAGACCGATGAAGCGTTCTTC
RF/R- TSC09	CAATGATGACACGCCAAGTAC
LF/F- TSC11	CTTGCTCAAGTCCTTGTTGTG
LF/R- TSC11	TATTCACGAACGAAAATCGATATACTTTCATCTTAGTTTCTC
RF/F- TSC11	AACAATAAACCCTTGCATAACGGTTGGTTATGATGATATAAT ATTTTC
RF/R- TSC11	CTTCTTTCTCGTTATCTTCATCGC
TCS05-Fusion-F	GAC<u>GCATGC</u>GTAACATCGGTATGGGAATCAAG
TCS05-Fusion-R	GCG<u>GGATCC</u>GAAACTCCTCCTTATTAAA
TCS03-Fusion-F	GAC<u>GCATGC</u>AGGAGCGGGTTGAAGATATG
TCS03-Fusion-R	GCG<u>GGATCC</u>CAAGCCCAAACGGACCA
TCS09-Fusion-F	GAC<u>GCATGC</u>CTTTGAGAAACCTGTGGC
TCS09-Fusion-R	GCG<u>GGATCC</u>CCTTGTCTTACCAGATATTC
SPD0344RTF	GCAGAAAAATTGAGCCGAAC
SPD0344RTR	CGAAATACGCGCTACCAGAT
SPD0144RTF	GGCGAGAAAGCTTAAGCAGA
SPD0144RTR	TTGTGCCCAAACTCATCAAA
SPD0939RTF	CAAAATTGAAAAATGGGGCTA
SPD0939RTR	GCAAGCTGAGAGACAATCTGC
SPD0063RTF	GCAAGCTGAGAGACAATCTGC
SPD0063RTR	CGGTTCAGGTCTTTTGGTA
SPD0065RTF	GGACCTCTTTGTAACAGGAA
SPD0065RTR	CATCTGCCAATTCCTTAGGA
SPD0420RTF	TGGTGTTTACGCACGTCTTG
SPD0420RTR	CATCAACCCCGTAAAGGTCAC
SPD0709RTF	TCGTGTGGCTGCCAAGCGTG
SPD0709RTR	GGCTGATCCACCAGCTGAGTC
SPD0722RTF	CGTCACCTTCACATGACACC
SPD0722RTR	CATGTTGAATGCTCCGTCAC
SPD1499RTF	GGAGTGAGCCAATTTTTGC
SPD1499RTR	GCAGGCATAACATCAGCT
SPD1504RTF	AGCAACCTCTGGCAAATGAA
SPD1504RTR	ATAGTAATCTCTTGGAATT
SPD1634RTF	TCTCGGTGCTCGTATGACAG
SPD1634RTR	CACCTGCAACTTCAGCGATA
SPD1652RTF	CTTTGGTGCCAAATCTCGTT
SPD1652RTR	GCAAGGGTACGGTTGATGAC
